# Temporal and spatiotemporal autocorrelation of daily concentrations of *Alnus, Betula*, and *Corylus* pollen in Poland

**DOI:** 10.1007/s10453-014-9354-2

**Published:** 2014-11-15

**Authors:** J. Nowosad, A. Stach, I. Kasprzyk, Ł. Grewling, M. Latałowa, M. Puc, D. Myszkowska, E. Weryszko- Chmielewska, K. Piotrowska-Weryszko, K. Chłopek, B. Majkowska-Wojciechowska, A. Uruska

**Affiliations:** 1 Institute of Geoecology and Geoinformation, Adam Mickiewicz University, Dzięgielowa 27, 61-680 Poznań, Poland; 2 Department of Environmental Biology, University of Rzeszów, Zelwerowicza 4, 35-601 Rzeszów, Poland; 3 Laboratory of Aeropalynology, Faculty of Biology, Adam Mickiewicz University, Umultowska 89, 61-614 Poznań, Poland; 4 Department of Plant Ecology, University of Gdańsk, Wita Stwosza 59, 80-308 Gdańsk, Poland; 5 Department of Botany and Nature Conservation, University of Szczecin, Felczaka 3c, 71-412 Szczecin, Poland; 6 Department of Clinical and Environmental Allergology, Jagiellonian University Medical College, Śniadeckich 10, 31-531 Kraków, Poland; 7 Department of Botany, University of Life Sciences in Lublin, Akademicka 15, 20-950 Lublin, Poland; 8 Department of General Ecology, University of Life Sciences in Lublin, Akademicka 15, 20-950 Lublin, Poland; 9 Faculty of Earth Sciences, University of Silesia, Będzińska 60, 41-200 Sosnowiec, Poland; 10 Department of Immunology, Rheumatology and Allergy, Faculty of Medicine, Medical University, Pomorska 251, 92-215 Łódź, Poland

**Keywords:** Tree pollen, Allergenic pollen, Betulaceae, Diurnal variation, Temporal autocorrelation, Space–time autocorrelation

## Abstract

The aim of the study was to determine the characteristics of temporal and space–time autocorrelation of pollen counts of *Alnus*, *Betula*, and *Corylus* in the air of eight cities in Poland. Daily average pollen concentrations were monitored over 8 years (2001–2005 and 2009–2011) using Hirst-designed volumetric spore traps. The spatial and temporal coherence of data was investigated using the autocorrelation and cross-correlation functions. The calculation and mathematical modelling of 61 correlograms were performed for up to 25 days back. The study revealed an association between temporal variations in *Alnus*, *Betula*, and *Corylus* pollen counts in Poland and three main groups of factors such as: (1) air mass exchange after the passage of a single weather front (30–40 % of pollen count variation); (2) long-lasting factors (50–60 %); and (3) random factors, including diurnal variations and measurements errors (10 %). These results can help to improve the quality of forecasting models.

## Introduction

Measured at a point, an air pollen count is the result of many factors affecting the production and dispersion of pollen in the atmosphere. Hence, changing one or a few factors usually does not cause an immediate and abrupt change in pollen concentrations. Rather, the change is gradual and somewhat delayed. The same holds for its spatial dimension. Understanding the character of atmospheric pollen concentrations can greatly help to improve the quality of forecasting models. Therefore, it is important in the prophylaxis of pollen allergies.

Alder (*Alnus* Mill.), birch (*Betula* L.), and hazel (*Corylus* L.) belong to the order Fagales Engl. and the family Betulaceae Gray (Bremer et al. 2009). Those trees are important sources of allergenic pollen in the temperate climatic zone of the Northern Hemisphere (Stach et al. 2008). The major pollen allergens from members of the family Betulaceae are structurally and immunochemically similar. Therefore, alder, birch, and hazel allergens have a high degree of cross-reactivity (Mothes and Valenta 2004). Seasons with abundant hazel or alder pollen can lead to stronger reactions to birch pollen and extend the birch pollen season (Emberlin et al. 1997; Puc 2003; Rodriguez-Rajo et al. 2004). Approximately 15 % of the European population suffers from allergies, and Poland is one of the countries with the highest allergy incidence rates, up to 45 %. It mostly affects children and young people (Heinzerling et al. 2009; Piotrowska and Kubik-Komar 2012; Malkiewicz et al. 2013). Sensitisation rates to trees belonging to the family Betulaceae are high in Central/Western Europe, with Poland showing high sensitisation rates for alder (22.8 %), birch (27.7 %), and hazel (22.3 %) (Heinzerling et al. 2009). The number of pollen grains needed to provoke an allergic reaction in people depends on individual reactivity and differs according to species and region. In Poland, the first allergy symptoms are observed at $20\,\hbox {pollen/m}^{3}$ of air for birch, $35\,\hbox {pollen/m}^{3}$ of air for hazel, and $45\,\hbox {pollen/m}^{3}$ of air for alder (Rapiejko et al. 2007).

The pollen grain identification is conducted at the genus level. Each genus has a dominant species in Poland: *Alnus glutinosa*, *Betula pendula*, and less common *Betula pubescens*, *Corylus avellana*, and their cultivars. All species within a particular genus bloom at very similar times. Alder and hazel pollen is the first to appear in the air. The pollination period and the start of the pollen season are highly variable from year to year and depend on the rather unstable weather conditions in late winter and spring (Smith et al. 2007; Kaszewski et al. 2008; Hájková et al. 2009; Rodriguez-Rajo et al. 2009; Myszkowska et al. 2011). Alder and hazel shed their pollen from the end of January to mid-April, while the birch pollination period is relatively short and occurs in the second half of April and early May (Weryszko-Chmielewska et al. 2001; Kluza-Wieloch and Szewczak 2006; Puc 2007; Grewling et al. 2012a).

Pollination is affected by the geographical location and local climate. The phases and duration of pollen seasons as well as the skewness, kurtosis, and annual totals of pollen grains heavily depend on the geographical position (Myszkowska et al. 2010). Nevertheless, annual totals of alder and hazel pollen show different spatial patterns. For *Alnus*, the annual pollen total increases from east to west, and for *Corylus*, there is an increase in the northerly direction (Myszkowska et al. 2010). Furthermore, weather conditions such as mean air temperature, total precipitation, relative humidity, and wind direction and speed have an important effect on the duration and intensity of pollen release and concentration in the air. As a result, these variables can be used for constructing forecast models (Rodriguez-Rajo et al. 2009; Puc 2012).

Spatial and space–time autocorrelation is functionally important in many ecosystems. Pollen production varies in both space and time; therefore, documenting its patterns and understanding the causes of variations is important to aerobiology. Moreover, describing temporal autocorrelation (one taxon—one location) and space–time autocorrelation (one taxon—two/many locations) should be the first step in developing models of pollen concentrations. Nowadays, autocorrelation analysis is rarely used in aerobiological research (Rodriguez-Rajo et al. 2006), except for mast seeding studies. Liebhold et al. (2003) quantified within-population spatial synchrony in mast dynamics of North American oaks, Garrison et al. (2006) studied spatial synchrony and temporal patterns of acorn production in California black oaks, and Ranta and Satri (2007) analysed the synchronisation of high and low pollen years of *Betula*, *Alnus*, *Corylus*, *Salix,* and *Populus*. There has been no comprehensive analysis of alder, birch, and hazel temporal and space–time synchrony so far.

The main objective of the present study was to determine mean multi-year characteristics of temporal and space–time autocorrelation of the pollen counts of *Alnus*, *Betula,* and *Corylus* in the air of eight cities in Poland.

## Materials and methods

### Study area

**Fig. 1:**
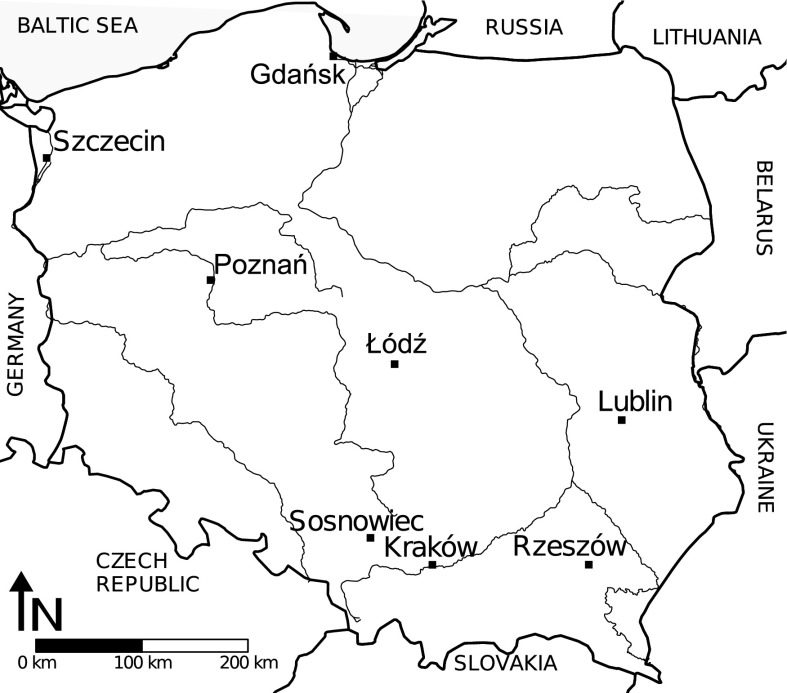
Sites used for the study of temporal and spatiotemporal autocorrelation of daily pollen concentrations in Poland

The monitoring of the concentrations of *Alnus*, *Betula*, and *Corylus* pollen in the air was conducted in Gdańsk, Kraków, Lublin, Łódź, Poznań, Rzeszów, Sosnowiec, and Szczecin (Fig. 1; Table 1). The studies covered 8 years of measurement (2001–2005 and 2009–2011) in Gdańsk, Kraków, Lublin, Poznań, Rzeszów, and Sosnowiec, 7 years in Szczecin (2002–2005 and 2009–2011), and 6 years in Łódź (2003–2005 and 2009–2011). The minimum distance between the locations was 75 km (Kraków–Sosnowiec), and the maximum one 640 km (Rzeszów–Szczecin) (Fig. 1).

**Table 1:** Characteristics of the study sites: latitude, longitude, and altitude of the aerobiological monitoring sites, area and population of the cities, and mean temperatures recorded at meteorological stations located in them

City	$\lambda$ (DD)	$\phi$ (DD)	Altitude (a.s.l.)	Area $(\hbox {km}^2)$	Population (in thousands)	Mean temperature $(^\circ \hbox {C})$ (1983–2012)				
						Annual	January	February	March	April
Gdańsk	18.6131	54.3856	10	262	460	7.42	−1.40	−1.40	1.78	6.66
Kraków	19.9559	50.0637	212	327	758	8.47	−1.90	−1.11	3.12	8.94
Lublin	22.5402	51.2437	198	147	348	7.81	−2.66	−2.22	1.93	8.31
Łódź	19.4748	51.7715	216	293	719	8.43	−1.62	−1.05	2.80	8.76
Poznań	16.9243	52.4671	91	262	551	8.96	−0.62	−0.18	3.59	9.08
Rzeszów	22.0160	50.0293	209	117	182	8.46	−2.10	−1.28	2.87	8.79
Sosnowiec	19.1389	50.2972	252	91	214	8.47	−1.50	−0.81	3.08	8.72
Szczecin	14.5478	53.4395	30	301	409	8.96	0.18	0.57	3.74	8.60

Poland is a country in Central Europe with an average elevation of 173 m and an area of 322,575 square kilometres (Dmochowska 2013). Only 3 % of the Polish territory is higher than 500 m. Despite its lowland character and a relatively small and compact area, Poland displays marked climatic differences. There is a NW–SE oceanic–continental gradient connected with the predominantly westerly circulation from the north Atlantic Ocean. In the analysed data set, this gradient is best represented by the locations of Szczecin and Rzeszów. This pattern is locally modified by the impact of the Baltic Sea and relief. At the locations concerned, the mean annual temperature ranges from 7.4 to $8.8\,^\circ \hbox {C}$, annual precipitation totals, from 500 to 750 mm, and the duration of the growing season, from 205 to 225 days (Lorenc 2005). The Kolmogorov–Smirnov two-sample test shows no significant difference (*D* = 0.12, *p* value = 0.14) between the daily temperature for 8 years of study (2001–2005 and 2009–2011) and a 30-year time series of measurement (1983–2012).

Poland has an average urbanisation level, with an urban population of about 62 % and artificial surfaces covering 4 % of the land (Dmochowska 2013). Various kinds of agricultural land cover 62.5 % of the country’s area, including arable land, permanent crops, pastures, and heterogeneous farmland. Predominant crops include potatoes, sugar beets, wheat, rye, and barley (Dmochowska 2013). Forests, mostly coniferous, occupy about 30 % of the country’s area. Their share is the largest in northern and western Poland (Milewski 2013).

### Aerobiological data

Daily average pollen concentrations of three taxa such as *Alnus*, *Betula,* and *Corylus* were analysed over 8 years (2001–2005 and 2009–2011), depending on the availability of data. The investigations were carried out using Hirst-designed volumetric spore traps (Hirst 1952). Ten litres per minute was the intake of air through the narrow inlet opening. Airborne particles attached themselves to an adhesive tape wrapped around a moving drum. The drum moved at a constant speed of two millimetres per hour, performing a complete turn during 1 week. Every week, the tape was cut into seven segments and microscopic slides were made. Pollen grains were counted from twelve vertical or four horizontal strips, and their sum was multiplied by a factor dependent on the surface of a slide (Frenguelli 2003). The results were expressed as a daily average number of pollen grains in $\hbox {m}^3$ of air sampled per 24 h (Comtois 1998).

### Statistical analysis

All calculations were carried out using the Variowin, ISATIS, and R software packages (Pannatier 1996; Bleines et al. 2011; R Core Team 2013). The limits of pollen seasons were calculated using the 90 % method (Nilsson and Persson 1981) whereby a season starts when 5 % of the total catch has been achieved and ends when 95 % has been reached.

Data of all years and particular site have been combined into a single table, in which *X* was the cumulative number of a day from 1 January 2001, and *Y*—the value of daily concentrations of taxon A in site B. In order to speed up calculations and eliminate the large number of lags with zero concentration values, the period from 1 to 180 days was taken into account each year.

Second-order properties, such as the mean, variance, and serial correlation, play a key role in the study of time-series data (Cowpertwait and Metcalfe 2010). The number of time steps between the variables is known as the lag. If the correlation between variables depends only on the lag, the variables may be correlated and the model is second-order stationary. Autocorrelation, also known as serial correlation, is a correlation of a variable with itself at different times. Consider a time-series model that is second-order stationary. The autocovariance function (*acvf*), $\gamma _{k}$, can be defined as a function of the lag *k*:

1 $$\begin{aligned} \gamma _{k} = E [(x_{t} - \mu )(x_{t+k} - \mu )] \end{aligned}$$

The function $\gamma _{k}$ does not depend on *t* because the expectation is the same at all times *t*. The lag *k* autocorrelation function (*acf*), $\rho _{k}$, is

2 $$\begin{aligned} \rho _{k} = \frac{\gamma _{k}}{\sigma ^{2}} \end{aligned}$$

The estimate of autocovariance $\textit{c}_{k}$ is defined as:

3 $$\begin{aligned} \textit{c}_{k} = \frac{1}{n}\sum _{t=1}^{n-k} (x_{t} - \bar{x})(x_{t+k} - \bar{x}) \end{aligned}$$

where $\bar{x}$ is the sample mean of the time series.

The most satisfactory estimate of the *k*th lag autocorrelation $\rho _{k}$ is

4 $$\begin{aligned} \textit{r}_{k} = \frac{\textit{c}_{k}}{\textit{c}_{0}} \end{aligned}$$

**Fig. 2:**
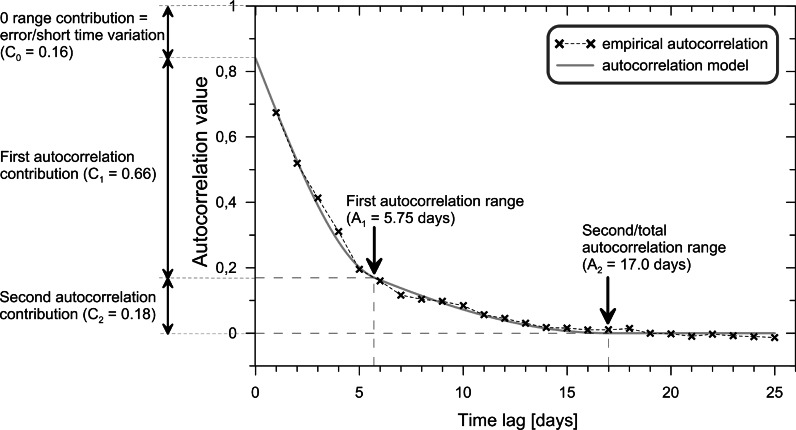
Explanation of the terms of a correlogram and its mathematical model

The $\textit{r}_{k}$ values can be called the sample autocorrelation function. All sample autocorrelations lie between $-1$ and 1. A plot of the correlation function against the lag is referred to as a correlogram (Fig. 2).

Consider a time-series model for variables *x* and *y* that are stationary in the mean and the variance. If the serial correlations of those variables and their correlations with each other at different time lags depend only on a lag, the combined model is second-order stationary. Therefore, a cross-covariance function (*ccvf*), $\gamma _{k}(x,y)$, can be defined as a function of the lag, *k*:

5 $$\begin{aligned} \gamma _{k}(x,y) = E [(x_{t+k} - \mu _{x})(y_{t} - \mu _{y})] \end{aligned}$$

The lag *k* cross-correlation function (*ccf*), $\rho _{k}(x,y)$ is

6 $$\begin{aligned} \rho _{k}(x,y) = \frac{\gamma _{k}(x,y)}{\sigma _{x}\sigma _{y}} \end{aligned}$$

**Fig. 3:**
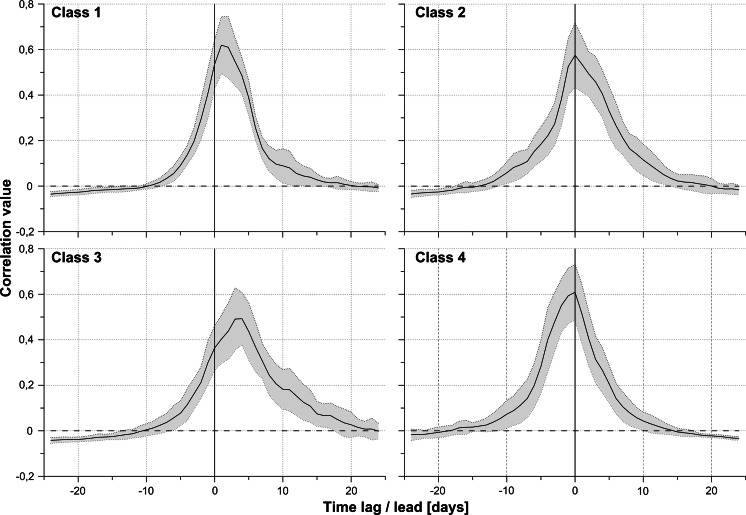
Average cross-correlograms and their standard deviations for individual classes

The cross-correlation function, in contrast to the autocorrelation function, is not symmetric (Fig. 3). The estimate *ccvf*, $\textit{c}_{k}(x,y)$ is defined as:

7 $$\begin{aligned} \textit{c}_{k}(x,y) = \frac{1}{n}\sum _{t=1}^{n-k} (x_{t+k} - \bar{x})(y_{t} - \bar{y}) \end{aligned}$$

The sample *ccf* is calculated as:

8 $$\begin{aligned} \textit{r}_{k}(x,y) = \frac{\textit{c}_{k}(x,y)}{\sqrt{\textit{c}_{0}(x,x)\textit{c}_{0}(y,y)}} \end{aligned}$$

The *ccf* function can be shown as a plot of $\textit{r}_{k}(x,y)$ against *k*, which is called the cross-correlogram.

The zero structure $(C_{0})$ on the correlogram plot (Fig. 2) is represented by an extrapolated point of intersection between the correlogram model and the y-axis. It gives the estimated value of autocorrelation at the 'zero' interval. The results of two measurements made at the same place and at the same time should be identical, which means that the curve of the model should touch the *y*-axis where the correlation coefficient is equal to 1. However, usually this is not so: only 6 out of the 61 estimated *r* values for the 0-day interval equal 1 $(C_{0}=0.0)$; see Table 2. The source of the differences is measurement errors and the temporal variability of pollen concentrations during the sampling period, i.e. within 1 day. Therefore, the $C_{0}$ structure in Fig. 2 and Tables 2 and 3 is referred to as 'error/short time variation'.

Typically, an empirical correlogram does not steadily decrease from the maximum to zero. Sometimes, it is composed of several segments with a different inclination. The connections of the segments show distinct changes in the shape of the curve. This characteristic of a temporal correlogram is interpreted as a result of the impact of factors/processes operating at different scales (intervals), for example a single event, a diurnal cycle, and a seasonal cycle. Mathematical correlogram modelling allows an estimation of the length (time range) of its individual segments, and this provides a basis for reasoning about their origins. Theoretical basis of this methodology is presented in geostatistical textbooks, inter alia, Goovaerts (1997) and Chiles and Delfiner (2012).

The calculation of 61 correlograms (8 locations $\times$ 3 taxa $\times$ 2 or 3 variants with excluding extremes) and 84 cross-correlograms (28 pairs of locations $\times$ 3 taxa $\times$ 1 variant) as well as their mathematical modelling were performed for up to 25 days back. This process consisted of three stages. Sample correlograms and cross-correlograms were calculated at different lags. Next, parametric functions were identified using one of the conditionally negative definite functions, such as the Gaussian, the exponential and the spherical model, or by nesting these models. Finally, sample correlograms were fitted to the parametric form by adjusting model parameters: the sill, nugget, and range. The best fit was obtained by minimising the indicative goodness-of-fit (IGF) criterion (Pannatier 1996).

The mathematical modelling of the correlograms was intended to smooth random fluctuations of the empirical data, to interpolate and extrapolate the data, and to objectively assess the range and share of individual structures.

Since all the measurement series exhibited highly skewed distributions with a predominance of low and average values and single, far outlying extreme values, the autocorrelation analysis was conducted twice: for the entire set and with the extremes eliminated. For the determination of extreme values, two threshold values were used. The first was put at the place of disruption of the frequency histogram (selection 1), the other cut off the data outliers (selection 2) (Fig. 5).

The Pearson's coefficient of product–moment correlation between all the cross-correlograms was calculated. Next, based on the *r* value, a hierarchical cluster analysis using Ward’s method was carried out (Ward Jr 1963). Finally, on the basis of a plot of linkage distances across steps, the cross-correlograms were divided into four classes (Fig. 3).

**Fig. 4:**
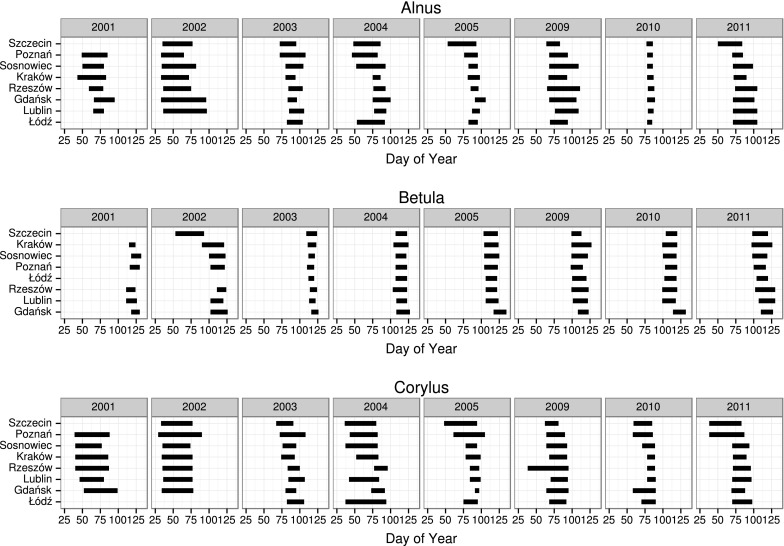
Length of the pollen season for the same taxa in particular years at various locations. The sites are ordered by the average starting date of the pollen season

## Results

### Data characteristics

The mean annual alder, birch, and hazel pollen concentrations were 2,952.67, 7,657.00, and 435.65 grains, respectively. The deposition of pollen of those taxa differed widely between particular years and measurement sites. The annual alder pollen totals ranged from 429.2 grains (Sosnowiec in 2009) to 13,582.2 (Poznań in 2010). The annual birch pollen counts varied from 1,182.6 (Rzeszów in 2009) to 34,095 (Lublin in 2003), and hazel pollen from 68.4 (Gdańsk in 2005) to 1,267.2 (Kraków in 2010).

The start of an average *Alnus* pollen season was on the 67th day of the year (March 8) (SD = 16.08 days), and its average duration was 26 days. The earliest start of the *Alnus* season occurred on 2 February 2004 in Kraków, and the latest, on 1 April 2005 in Gdańsk. The *Betula* season started on the 105th day of the year (April 15) (SD = 5.58 days) and lasted 17.5 days on the average. The Gdańsk results differed from the other sites, with an average birch pollen season starting here on April 22 (112th day of the year). The average duration of the *Corylus* pollen season was about 30 days, with the season starting on the 60th day of the year (March 1) (SD = 17.73 days). Variations in the duration of the pollen season depended not only on the taxon, but also on the location. The standard deviation of the duration value for alder, birch, and hazel was 13.33, 5.77, and 13.31 days, respectively (Fig. 4).

The descriptive statistics of daily pollen concentrations were calculated for the pollen seasons. Pollen concentration data were characterised by a strongly right-skewed frequency distribution. The median values of 19.0 (SD = 267.62), 133.0 (SD = 845.05), and 4.7 (SD = 30.94) grains per cubic metre of air represented *Alnus*, *Betula*, and *Corylus*, respectively. Pollen counts differed widely among years and sites. The lowest pollen count median for all data was in 2004, at 9.0, and the highest in 2010, at 124.0. The highest medians of daily pollen concentrations were recorded in Szczecin (*Alnus*—53.0) and Lublin (*Betula*—279.2, *Corylus*—13.0), and the lowest ones in Sosnowiec (*Alnus* - 10.0), Kraków (*Betula*—66.0), and Poznań (*Corylus*—1.0).

### Temporal autocorrelation

 Autocorrelation coefficients of the analysed taxa with a 1-day lag were calculated (Table 2).

**Table 2:** Parameters of correlogram models of pollen counts

Taxa	City	Data subset	$C_{0}$	T1	$C_{1}$	$A_{1}$	T2	$C_{2}$	$A_{2}$	T3	$C_{3}$	$A_{3}$	$r-1$ day
*Alnus*	Gdańsk	All data	0.20	Sph	0.59	2.50	Sph	0.21	11.00				0.471
*Alnus*	Gdańsk	Selection 1	0.15	Exp	0.58	5.00	Sph	0.24	24.00				0.565
*Alnus*	Gdańsk	Selection 2	0.13	Exp	0.44	3.25	Sph	0.18	10.50	Sph	0.20	20.25	0.565
*Alnus*	Kraków	All data	0.05	Sph	0.48	6.50	Sph	0.48	11.75				0.772
*Alnus*	Kraków	Selection 1	0.10	Sph	0.30	3.00	Sph	0.61	22.50				0.729
*Alnus*	Kraków	Selection 2	0.07	Sph	0.26	2.75	Sph	0.21	11.50	Sph	0.48	19.25	0.741
*Alnus*	Lublin	All data	0.06	Sph	0.56	4.75	Sph	0.38	10.25				0.696
*Alnus*	Lublin	Selection 1	0.08	Sph	0.25	2.50	Sph	0.16	7.50	Sph	0.49	15.75	0.722
*Alnus*	Lublin	Selection 2	0.01	Sph	0.35	2.80	Sph	0.22	8.50	Sph	0.42	14.00	0.751
*Alnus*	Łódź	All data	0.10	Sph	0.91	8.50							0.761
*Alnus*	Łódź	Selection 1	0.00	Sph	0.26	3.50	Sph	0.74	10.50				0.776
*Alnus*	Poznań	All data	0.00	Sph	0.33	4.75	Sph	0.48	9.75	Sph	0.20	15.00	0.835
*Alnus*	Poznań	Selection 1	0.15	Sph	0.27	4.75	Sph	0.58	15.25				0.709
*Alnus*	Rzeszów	All data	0.07	Sph	0.28	4.00	Sph	0.65	8.20				0.709
*Alnus*	Rzeszów	Selection 1	0.04	Sph	0.55	3.25	Sph	0.39	15.25				0.685
*Alnus*	Rzeszów	Selection 2	0.11	Sph	0.31	3.00	Sph	0.56	10.25				0.659
*Alnus*	Sosnowiec	All data	0.04	Sph	0.46	5.25	Sph	0.51	11.00				0.750
*Alnus*	Sosnowiec	Selection 1	0.02	Sph	0.18	4.25	Exp	0.80	13.50				0.725
*Alnus*	Sosnowiec	Selection 2	0.00	Exp	0.37	6.50	Sph	0.62	10.25				0.789
*Alnus*	Szczecin	All data	0.09	Sph	0.30	5.00	Sph	0.62	17.75				0.722
*Alnus*	Szczecin	Selection 1	0.17	Sph	0.16	3.00	Sph	0.66	22.00				0.666
*Alnus*	Szczecin	Selection 2	0.09	Sph	0.24	4.75	Sph	0.67	17.50				0.740
*Betula*	Gdańsk	All data	0.09	Sph	0.26	2.75	Sph	0.41	6.25	Sph	0.26	16.25	0.652
*Betula*	Gdańsk	Selection 1	0.02	Sph	0.32	2.50	Sph	0.32	6.75	Sph	0.36	16.50	0.706
*Betula*	Kraków	All data	0.10	Sph	0.15	2.25	Sph	0.16	10.75	Sph	0.61	20.50	0.740
*Betula*	Kraków	Selection 1	0.13	Sph	0.14	2.50	Sph	0.12	13.50	Sph	0.66	31.00	0.725
*Betula*	Kraków	Selection 2	0.04	Sph	0.21	3.25	Sph	0.22	10.75	Sph	0.58	27.00	0.800
*Betula*	Lublin	All data	0.18	Sph	0.25	2.25	Sph	0.27	10.00	Sph	0.32	17.00	0.600
*Betula*	Lublin	Selection 1	0.05	Sph	0.15	2.50	Sph	0.20	9.75	Sph	0.65	22.25	0.794
*Betula*	Łódź	All data	0.00	Sph	0.49	2.75	Sph	0.52	11.75				0.686
*Betula*	Łódź	Selection 1	0.07	Sph	0.16	2.25	Sph	0.33	10.75	Sph	0.47	20.25	0.748
*Betula*	Łódź	Selection 2	0.08	Sph	0.21	2.75	Sph	0.29	9.25	Sph	0.45	19.50	0.664
*Betula*	Poznań	All data	0.03	Sph	0.23	2.25	Sph	0.24	9.75	Sph	0.52	17.50	0.748
*Betula*	Poznań	Selection 1	0.06	Sph	0.22	2.25	Sph	0.18	9.75	Sph	0.57	20.00	0.744
*Betula*	Rzeszów	All data	0.10	Sph	0.25	2.75	Sph	0.20	6.75	Sph	0.45	18.00	0.647
*Betula*	Rzeszów	Selection 1	0.10	Sph	0.12	3.00	Sph	0.22	8.75	Sph	0.57	21.80	0.756
*Betula*	Rzeszów	Selection 2	0.14	Sph	0.12	3.00	Sph	0.17	8.75	Sph	0.61	24.00	0.681
*Betula*	Sosnowiec	All data	0.02	Exp	0.40	2.75	Sph	0.29	8.50	Sph	0.30	13.75	0.578
*Betula*	Sosnowiec	Selection 1	0.03	Sph	0.22	3.75	Sph	0.26	6.75	Sph	0.50	17.50	0.767
*Betula*	Szczecin	All data	0.19	Sph	0.24	4.50	Sph	0.58	16.50				0.678
*Betula*	Szczecin	Selection 1	0.00	Sph	0.20	2.50	Sph	0.19	9.50	Sph	0.63	19.25	0.822
*Corylus*	Gdańsk	All data	0.05	Exp	0.65	4.75	Sph	0.29	12.50				0.657
*Corylus*	Gdańsk	Selection 1	0.36	Sph	0.21	3.75	Sph	0.41	20.75				0.492
*Corylus*	Kraków	All data	0.05	Sph	0.34	3.00	Sph	0.27	13.75	Sph	0.36	22.50	0.752
*Corylus*	Kraków	Selection 1	0.05	Sph	0.21	4.25	Sph	0.29	15.25	Sph	0.48	24.50	0.813
*Corylus*	Lublin	All data	0.15	Sph	0.31	2.25	Sph	0.29	9.00	Sph	0.25	16.75	0.597
*Corylus*	Lublin	Selection 1	0.18	Exp	0.21	3.50	Sph	0.57	21.50				0.653
*Corylus*	Lublin	Selection 2	0.10	Sph	0.26	2.50	Sph	0.22	15.00	Sph	0.42	22.25	0.713
*Corylus*	Łódź	All data	0.22	Sph	0.31	2.50	Sph	0.29	9.50	Sph	0.18	18.25	0.540
*Corylus*	Łódź	Selection 1	0.14	Sph	0.36	2.25	Sph	0.22	11.00	Sph	0.27	20.00	0.604
*Corylus*	Poznań	All data	0.16	Sph	0.21	3.00	Sph	0.64	17.75				0.682
*Corylus*	Poznań	Selection 1	0.07	Sph	0.37	3.50	Sph	0.56	17.75				0.685
*Corylus*	Rzeszów	All data	0.03	Sph	0.66	3.75	Sph	0.31	14.25				0.697
*Corylus*	Rzeszów	Selection 1	0.13	Sph	0.45	2.50	Sph	0.39	16.00				0.590
*Corylus*	Rzeszów	Selection 2	0.00	Sph	0.68	3.50	Sph	0.32	15.00				0.704
*Corylus*	Sosnowiec	All data	0.09	Sph	0.46	4.25	Sph	0.44	12.75				0.655
*Corylus*	Sosnowiec	Selection 1	0.09	Sph	0.25	2.25	Sph	0.18	8.00	Sph	0.40	17.25	0.643
*Corylus*	Sosnowiec	Selection 2	0.05	Exp	0.50	4.25	Sph	0.44	14.25				0.635
*Corylus*	Szczecin	All data	0.20	Sph	0.28	4.25	Sph	0.53	20.75				0.648
*Corylus*	Szczecin	Selection 1	0.18	Sph	0.22	3.75	Sph	0.60	22.25				0.671
*Corylus*	Szczecin	Selection 2	0.16	Sph	0.24	3.25	Sph	0.61	23.00				0.674

$C_{0}$—nugget component (error/short time variation), function type—T1 (type 1), T2 (type 2), T3 (type 3): Sph—Spherical, Exp—Exponential, time span of individual structures in days ($\hbox {A}_{1}$, $\hbox {A}_{2}$ and $\hbox {A}_{3}$), their share in total variability ($C_{1}$, $C_{2}$ and $C_{3}$), and the correlation coefficient with a 1-day delay (*r* $-1$ day) for distinguished taxons (*Alnus*, *Betula*, *Corylus*), cities (Gdańsk, Kraków, Lublin, Łódź, Poznań, Rzeszów, Sosnowiec, Szczecin), and subsets of data (all data and data with extremes eliminated—selection 1 and selection 2)

The average autocorrelation of daily counts was 0.68 with a range of 0.47–0.84. The autocorrelation figures of daily pollen counts with a 1-day lag were similar in the individual taxa. The average autocorrelation of daily *Alnus*, *Betula*, and *Corylus* pollen counts with a 1-day lag was 0.71 (range 0.47–0.84), 0.71 (range 0.58–0.75), and 0.65 (range 0.54–0.75), respectively.

**Table 3:** Descriptive statistics of parameters of correlogram models

Data subset	$C_{0}$		$C_{1}$		$C_{2}$		$C_{3}^a$		$\hbox {A}_{1}$		$\hbox {A}_{2}$		$\hbox {A}_{3}^a$	
	Mean	SD	Mean	SD	Mean	SD	Mean	SD	Mean	SD	Mean	SD	Mean	SD
All data	0.09	0.07	0.39	0.18	0.39	0.15	0.35	0.14	3.80	1.55	11.75	3.65	17.55	2.53
Selection 1	0.10	0.08	0.27	0.12	0.38	0.20	0.50	0.12	3.18	0.83	14.10	5.63	20.50	4.17
Selection 2	0.08	0.05	0.32	0.15	0.36	0.19	0.45	0.13	3.50	1.10	12.65	4.18	20.89	4.11

$C_{0}$—nugget component (error/short time variation), share in total variability ($C_{1}$, $C_{2}$ and $C_{3}$), and time span of individual structures in days ($A_{1}$, $A_{2}$ and $A_{3}$) for distinguished subsets of data (all data and data with extremes eliminated—selection 1 and selection 2)

$^a\,$Third structure was present in 29 of 61 correlogram models (47.5 %)

**Fig. 5:**
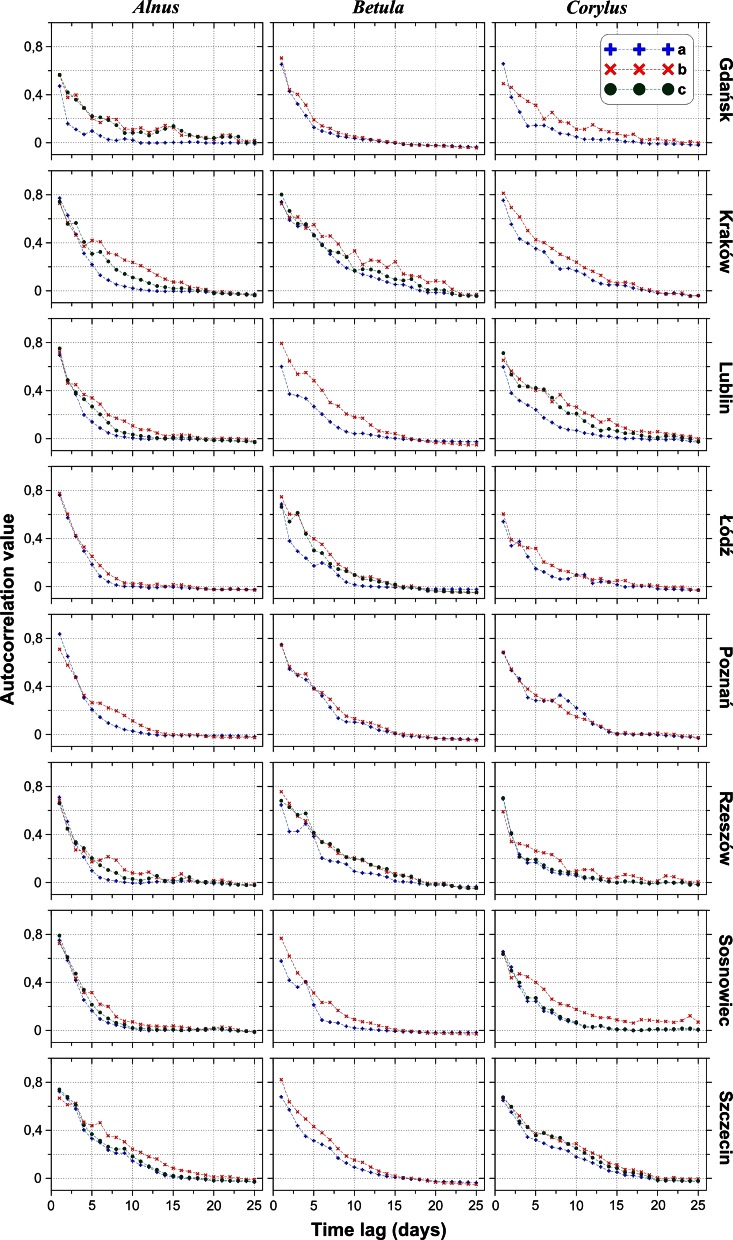
Matrix of sample (experimental) correlogram plots for the entire data set (*a*) and after the elimination of extreme figures (*b*-first threshold, *c*-second threshold) for the individual locations (*rows*) and taxa (*columns*)

**Fig. 6:**
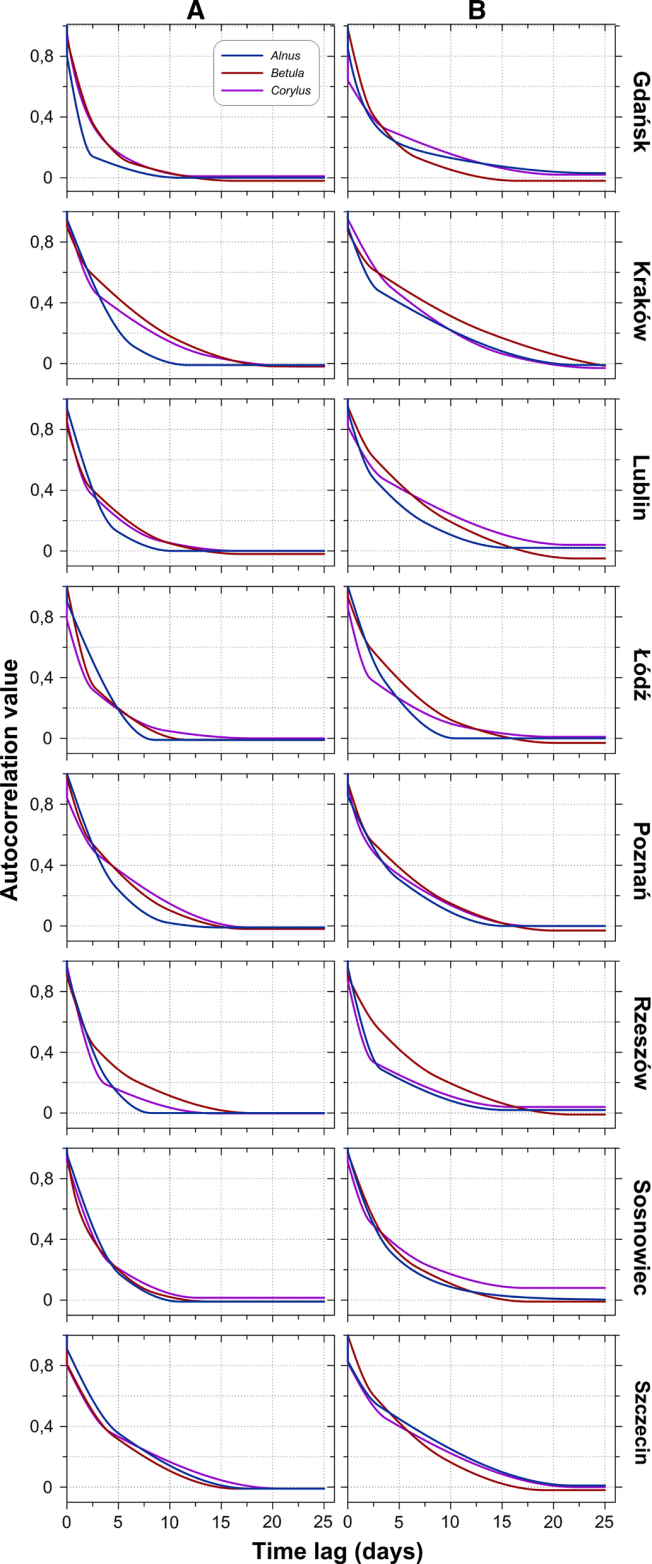
Matrix of correlogram models (**a**—all data, **b**—first threshold) for various taxa at the same locations

On average, correlogram shows a decline for the 3.5 days lag (*Alnus*—5.2, *Betula*—2.9, *Corylus*—3.5). Afterwards, the value kept decreasing steadily. Thus, autocorrelation coefficients reduced to zero after an average of 15.0 days (range 8.2–22.5) (Figs. 5, 6; Table 2). The removal of extreme values increased autocorrelation (0.70 on average) and expanded its range (19.5 and 18.2 days on average) (Tables 2, 3).

At least two individual structures were found in 60 out of the 61 correlogram models. A third structure was identified for 29 correlogram models (47.5 %): 5 out of 22 for *Alnus* (22.7 %), 17 out of 19 for *Betula* (89.5 %), and 7 out of 20 for *Corylus* (35.0 %) (Tables 2, 3).

### Space–time autocorrelation

**Fig. 7:**
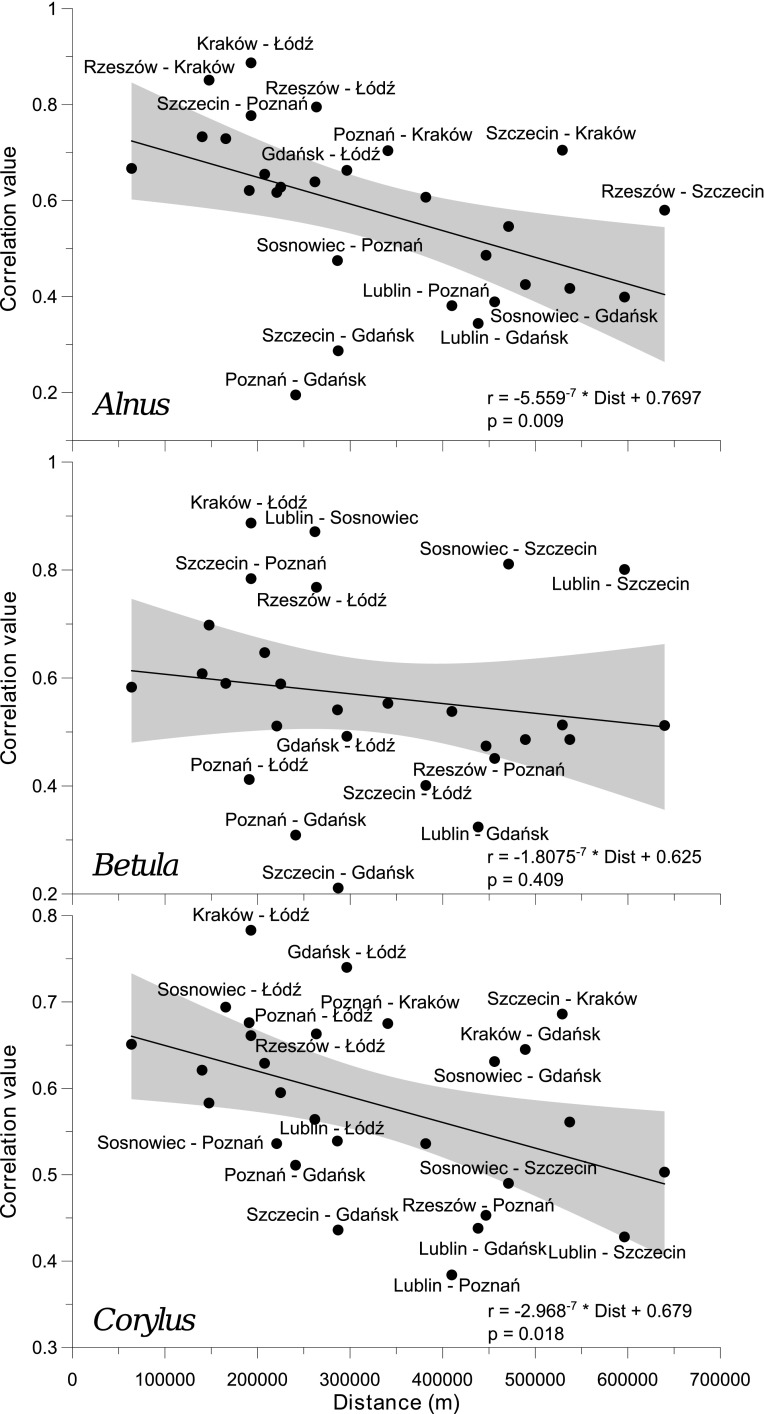
Correlation of concentrations of individual pollen taxa on the same day between the locations as a function of distance. The diagrams present linear regression curves and their 95 % confidence intervals (*shaded*), formulae for the models employed, and the significance level (*p* value) of functions. Only outlying pairs of stations are labelled

The correlation of individual pollen counts between the monitoring sites as a function of distance was shown in Figs. 7 (the same day) and 8 (with a 1-day offset). Few sites with regimes of pollen counts different to the remaining were identified. Pollen concentrations in Gdańsk were notably distinctive, especially to those noted in Szczecin, Poznań, and Lublin. Also, the correlation between some pairs of the cities was higher than others, especially in Kraków–Łódź (*Alnus, Betula*, and *Corylus* pollen), Lublin–Sosnowiec, Szczecin–Sosnowiec (*Betula* pollen), Gdańsk–Łódź, and Szczecin–Kraków (*Corylus* pollen).

**Fig. 8:**
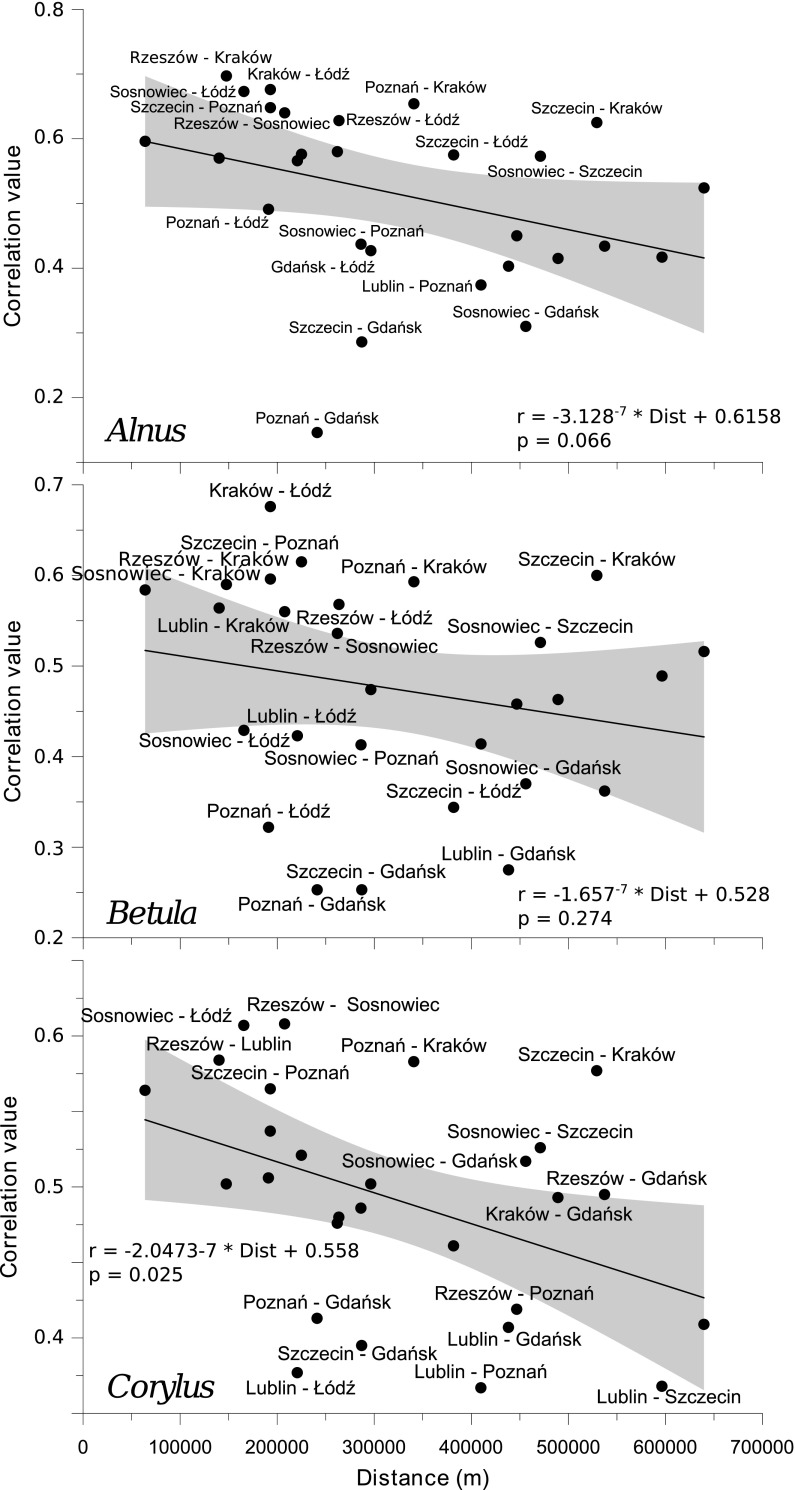
Correlation of concentrations of individual pollen taxa with a 1-day lag between the locations as a function of distance. The diagrams present linear regression curves and their 95 % confidence intervals (*shaded*), formulae for the models employed, and the significance level (*p* value) of functions. Only outlying pairs of stations are labelled

Correlation coefficients of 28 individual pairs of locations were calculated for each taxon. The average value was 0.58 (range 0.20–0.89). Correlation coefficients varied from 0.566 to 0.583. Furthermore, they were clearly, though not very strongly, dependent on the distance between pairs of monitoring sites (Fig. 7). With a 1-day lag, the correlation coefficients dropped to 0.49 (range 0.15–0.70) but the impact of distance still existed. The Kolmogorov–Smirnov two-sample test showed no significant differences between correlation coefficients of pairs of the observed taxa (*Alnus*–*Betula*, *Alnus*–*Corylus*, and *Betula*–*Corylus*).

**Fig. 9:**
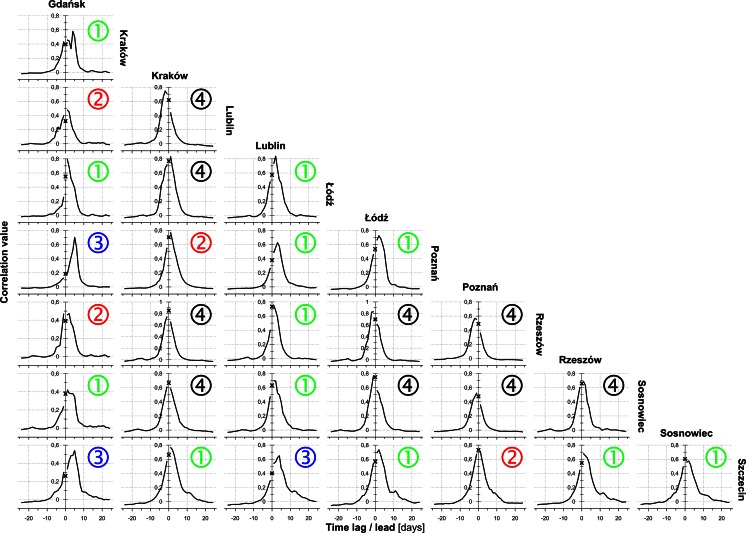
Diagrams of *Alnus* cross-correlation between locations with lead (*left branch*) and lag (*right branch*) time using all the data. The *black cross* shows the synchronous correlation value (lag/lead = 0 days). A comparison of these charts allows capturing the asymmetric relationship of time-predominant leads or delays. Cross-correlograms are labelled by class number (see Fig. 3 and description on page 5)

**Fig. 10:**
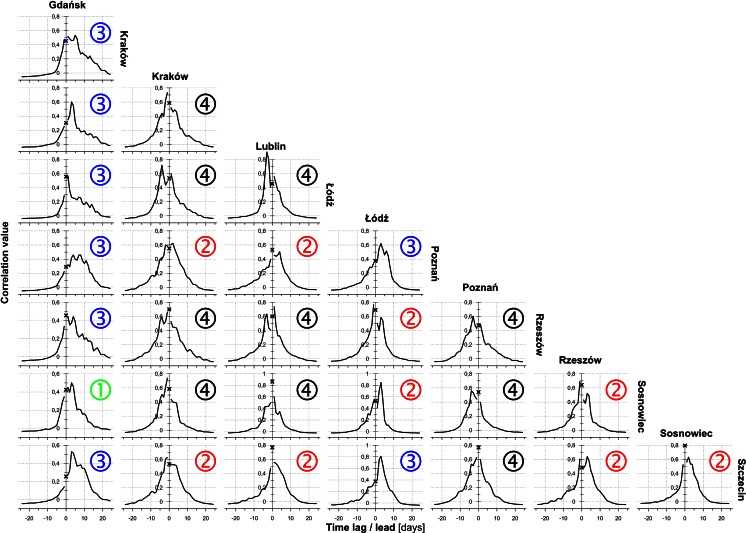
Diagrams of *Betula* cross-correlation between locations with lead (*left branch*) and lag (*right branch*) time using all the data. The *cross* shows the synchronous correlation value (lag/lead = 0 days). A comparison of these charts allows capturing the asymmetric relationship of time-predominant leads or delays. Cross-correlograms are labelled by class number (see Fig. 3 and description on page 5)

**Fig. 11:**
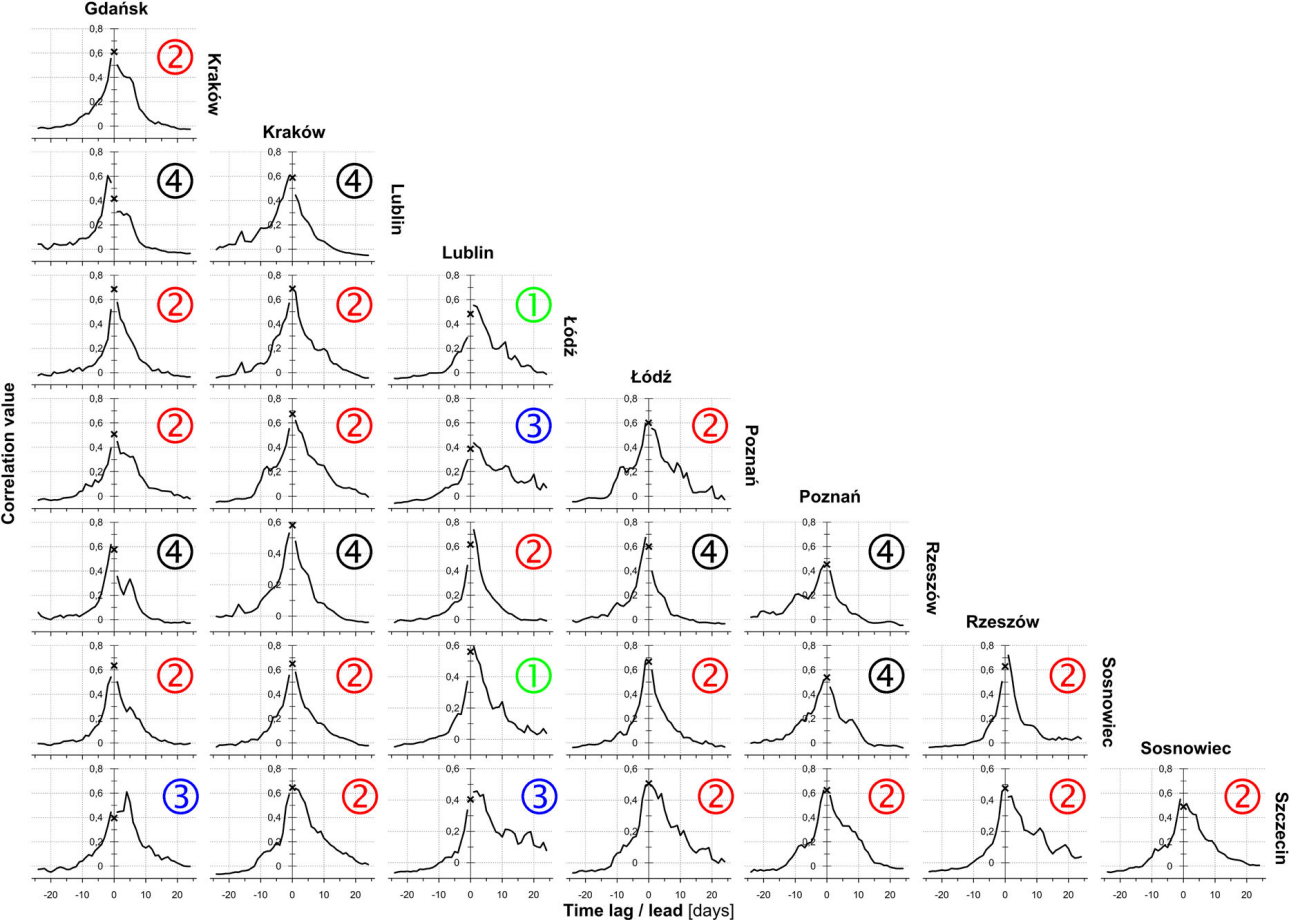
Diagrams of *Corylus* cross-correlation between locations with lead (*left branch*) and lag (*right branch*) time using all the data. The *cross* shows the synchronous correlation value (lag/lead = 0 days). A comparison of these charts allows capturing the asymmetric relationship of time-predominant leads or delays. Cross-correlograms are labelled by class number (see Fig. 3 and description on page 5)

Figures 9, 10, 11 show cross-correlograms of *Alnus*, *Betula*, and *Corylus* pollen counts in Poland. Cross-correlograms of alder were the most homogeneous, with symmetric and simple shapes. Moreover, the temporal range of pollen counts was the shortest, in most cases it did not exceed 10 days. Most of birch and hazel cross-correlograms had an asymmetrical shape, often with a few oscillations. Its range was generally longer, sometimes even more than 20 days. Furthermore, correlation rose along with a 1- to 5-day lag/lead in numerous pairs of monitoring sites. The strongest variation was seen in the case of Gdańsk. Most of its cross-correlograms were asymmetric and showed considerable variation.

The average cross-correlograms and their standard deviations for individual classes was shown in Fig. 3. In classes 2 and 4, the maximum correlation occurs on the same day (zero lag). They differ in a rate of correlation decrease in the direction of leads and delays. Their shapes are almost a reflection symmetry. Class 1 has a symmetric shape with a maximum delayed by 1 day. Class 3 has the lowest maximum correlation (0.5), the largest shift (lag of 4–5 days), and the most asymmetric shape.

The Chi-square test was used to examine whether the cross-correlogram classes (Fig. 3) depended on the taxon or the location. It showed a statistically significant relationship between the classes and the taxa (*p* value $\simeq 0.0002$), and between the classes and the location (*p* value $\simeq 0.01$). The first class was mostly *Alnus* (80 % of the class), the second class—*Corylus* (55 %), and the third class—*Betula* (57 %). Only in the fourth class, no taxa were dominating. However, the fourth class differentiates between the locations. This class occurred mostly in Kraków (10 out of 52) and Rzeszów (11 out of 52), and very rarely in Gdańsk (2 out of 52) and Szczecin (1 out of 52). In the third class, differentiation between the locations also occurred, with zero or a few cases in Sosnowiec (0 out of 28), Kraków (1 out of 28), and Rzeszów (1 out of 28) (Table 4).

**Table 4:** Frequency of cross-correlation classes at each location

City	Class 1	Class 2	Class 3	Class 4
Gdańsk	4	6	9	2
Kraków	2	8	1	10
Lublin	6	4	4	7
Łódź	5	7	3	6
Poznań	2	8	4	7
Rzeszów	2	7	1	11
Sosnowiec	5	8	0	8
Szczecin	4	10	6	1

The locations were classified by the frequency of the occurrence of the cross-correlogram class (Figs.  3, 9, 10, 11). There were major similarities between Kraków and Rzeszów, and Lublin and Łódź. Three groups were distinguished, the first consisting of Kraków, Rzeszów, and Sosnowiec; the second of Lublin, Łódź, and Poznań; and the third of Gdańsk and Szczecin.

## Discussion

Local and regional flora and land-use substantially affect the pollen spectrum. However, pollen concentrations in the air depend on more factors, including the vegetation structure, ability of particular taxa to spread their pollen, and the topoclimate and weather conditions in the current and preceding years. Especially, the effect of temperature on the intensity of the pollen season is often stressed (Corden et al. 2002; Rasmussen 2002).

Most pollen studies have focused on changes through time; hence, patterns across space remain largely unknown (Grewling et al. 2012b; León Ruiz et al. 2012; Ziello et al. 2012). Little research has been devoted to a spatial analysis of tree pollen in Poland. Myszkowska et al. (2010) investigated the relationship between the geographical location and the dynamics of *Alnus* and *Corylus* pollen seasons in Poland. However, temporal and spatiotemporal autocorrelation has not been taken into account before.

The form of the correlograms provides an understanding of the temporal variations in *Alnus*, *Betula*, and *Corylus* pollen counts in Poland. Notable changes in the shapes of the correlograms suggest that variations may be associated with three main groups of factors. 10 % of pollen count variations can be due to random factors, including diurnal fluctuations and measurement errors (Tables 2, 3). The autocorrelation value dropped substantially after 3.5 days. Thus, approximately 30–40 % of the variation could be caused by an exchange of air masses after the passage of a single weather front. The remaining 50–60 % depends on longer-lasting factors, exceeding 4 days.

According to Kotas et al. (2013), the variability of most of strings with a single air mass type is between 1 and 3 days. The influx of a new air mass has the physical characteristics of which differ from the previous one. An increase in the correlation between pollen counts in pairs of monitoring sites is delayed by 1–3 days (Figs. 9, 10, 11). This relationship could be partly associated with the movement of atmospheric fronts and the distance between the sites.

It was also found that pollen characteristics in Gdańsk differ from the rest of the sites. An average birch pollen season starts in Gdańsk 6 days later than in the rest of the country. The Gdańsk cross-correlograms were heterogeneous and asymmetric. Therefore, concentrations of tree pollen there vary considerably and clearly differ from many of the other monitoring sites. The uniqueness of Gdańsk can be explained by its coastal location and the impact of the Baltic Sea on the local climate.

The size of pollen grains of the studied taxa is similar $(Alnus \hbox{-}22\,\upmu \hbox {m}\,\times \,34\,\upmu \hbox {m},\,Betula \hbox{-}18\,\upmu \hbox {m}\,\times \, 28\,\upmu \hbox {m},\,Corylus \hbox{-}18\,\upmu \hbox {m} \,\times \, 23\,\upmu \hbox {m})$ (Accorsi et al. 1991). They were found to be among the longest-occurring pollen grains in the air (Dyakowska 1937). Alder, birch, and hazel pollen grains in Poland have similar transport and dispersion conditions. However, an analysis of the cross-correlogram classes shows that the character and range of autocorrelation depend on the location and the taxon. Moreover, the correlation coefficients decrease as the distance between sites increases. As follows from the cross-correlograms classes (Fig. 3), there are three groups of locations connected with the local topography and climate. The first consists of the coastal cities of Gdańsk and Szczecin. Their climate is influenced by Atlantic air masses and the proximity of the Baltic Sea—winters are mild, summers are not very hot, and the weather often changes. The second group is mostly linked with lowlands (Poznań, Łódź) and highlands (Lublin). The last group embraces Kraków, Sosnowiec, and Rzeszów. Those cities are located in southern Poland, and their climate is influenced by the Carpathian Mountains.

The location of monitoring sites in Poland is not random. They are primarily situated in populous cities with aerobiological research facilities. Urban areas modify the climate of their regions as a result of local changes in the properties of the land surface and the atmosphere. Consequently, those areas have a higher temperature than the surroundings. They are often referred to as urban heat islands. The average temperature in large Polish cities is 1–1.2$^\circ$C higher than in the surrounding areas, and the growing seasons last longer (Szymanowski 2005). Jochner et al. (2012) suggest that in completely urbanised areas, phenological phases start 2.6–7.6 days in advance. Therefore, caution should be exercised when interpolating the results for areas between stations. In this study, data from eight Polish monitoring sites were used, with none in the south-western and north-eastern parts of the country. Myszkowska et al. (2010) suggested higher concentrations of *Alnus* pollen in the north-eastern region of Poland to be caused by its long-distance transport and the dominant westerly direction of wind. An analysis of data from those parts of Poland could reveal other relationships in variations of tree pollen.

## Conclusion

- Temporal variations in the *Alnus*, *Betula*, and *Corylus* pollen counts seem to be associated with three main groups of factors: (i) diurnal variability and measurement errors, (ii) an exchange of air masses after the passage of a single weather front (every 3.5 days), and (iii) longer-lasting factors
- Due to the recurrence of circulation patterns, an increase in the correlation between pollen counts in pairs of monitoring sites is delayed by 1–3 days
- The start, course, and intensity of the pollen season in Gdańsk differ from the rest of the studied sites, possibly due to its coastal location and the local climate
- Three groups of locations connected with the local topography and climate could be distinguished. The first consists of the coastal cities, the second group is linked with lowlands and highlands, and the last group embraces those cities located in southern Poland.
- It would be worthwhile to analyse the relationship between variations in pollen counts and atmospheric circulation patterns
